# Poly[[tetra­aqua­tris­(μ_3_-hexane-1,6-di­carboxyl­ato)diterbium(III)] 0.25-hydrate]

**DOI:** 10.1107/S1600536811007719

**Published:** 2011-03-09

**Authors:** Fei-Fei Li, Hui-Ju Zhang, Li-Na Zhang

**Affiliations:** a Department of Physics and Chemistry, Henan Polytechnic University, Jiaozuo, Henan 454000, People’s Republic of China

## Abstract

In the title terbium coordination polymer, {[Tb_2_(C_6_H_8_O_4_)_3_(H_2_O)_4_]·0.25H_2_O}_*n*_, the Tb^III^  atom is nine-coordinated, forming a TbO_9_ polyhedra. Furthermore, two symmetric TbO_9_ polyhedra share their edges, forming Tb_2_O_16_ dimers, which are linked by adipate bridges into a layered structure. Inter­molecular O—H⋯O hydrogen bonds link these layers into a three-dimensional network. One of the C atoms of the adipate ligand is disordered over two positions with site-occupancy factors of 0.622 (9) and 0.378 (9). The structure also contains a disordered mol­ecule of water of hydration, lying close to a special position, with partial occupancy.

## Related literature

For background to coordination polymers, see: Moulton & Zaworotko (2001 ▶); Wood & Thompson (2007 ▶). For the structures of rare earth*-*-adipate compounds, see: Dimos *et al.* (2002 ▶); Duan *et al.* (2004 ▶); Kim *et al.* (2004 ▶); Kiritsis *et al.* (1998 ▶). For isotypic La(III) and Dy(III) structures, see: Kim *et al.* (2004 ▶); Lill *et al.* (2005 ▶).
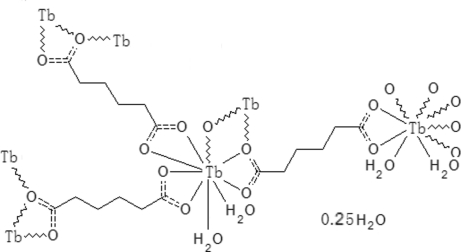


## Experimental

### 

#### Crystal data

[Tb_2_(C_6_H_8_O_4_)_3_(H_2_O)_4_]·0.25H_2_O
*M* *_r_* = 826.78
Monoclinic, 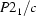

*a* = 11.603 (6) Å
*b* = 13.886 (7) Å
*c* = 8.969 (4) Å
β = 111.017 (7)°
*V* = 1348.9 (11) Å^3^
*Z* = 2
Mo *K*α radiation
μ = 5.27 mm^−1^
*T* = 298 K
0.25 × 0.05 × 0.05 mm

#### Data collection

Bruker APEXII CCD diffractometer
Absorption correction: multi-scan (*SADABS*; Sheldrick, 1996) ▶ *T*_min_ = 0.352, *T*_max_ = 0.779
7908 measured reflections
2335 independent reflections
2008 reflections with *I* > 2σ(*I*)
*R*_int_ = 0.037

#### Refinement

*R*[*F* ^2^ > 2σ(*F* ^2^)] = 0.025
*wR*(*F* ^2^) = 0.066
*S* = 1.06
2335 reflections
176 parameters
6 restraints
H-atom parameters constrained
Δρ_max_ = 0.89 e Å^−3^
Δρ_min_ = −1.85 e Å^−3^

### 

Data collection: *APEX2* (Bruker, 2008 ▶); cell refinement: *SAINT* (Bruker, 2008 ▶); data reduction: *SAINT*; program(s) used to solve structure: *SHELXS97* (Sheldrick, 2008 ▶); program(s) used to refine structure: *SHELXL97* (Sheldrick, 2008 ▶); molecular graphics: *SHELXTL* (Sheldrick, 2008 ▶); software used to prepare material for publication: *SHELXTL*.

## Supplementary Material

Crystal structure: contains datablocks I, global. DOI: 10.1107/S1600536811007719/pv2390sup1.cif

Structure factors: contains datablocks I. DOI: 10.1107/S1600536811007719/pv2390Isup2.hkl

Additional supplementary materials:  crystallographic information; 3D view; checkCIF report

## Figures and Tables

**Table 1:** Hydrogen-bond geometry (Å, °)

*D*—H⋯*A*	*D*—H	H⋯*A*	*D*⋯*A*	*D*—H⋯*A*
O8—H3⋯O4^i^	0.97	1.83	2.764 (4)	160
O8—H4⋯O5^ii^	0.92	1.78	2.691 (4)	170
O7—H1⋯O2^i^	0.91	1.75	2.657 (4)	170
O7—H2⋯O3^iii^	0.98	1.81	2.682 (4)	146

Symmetry codes: (i) 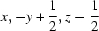; (ii) 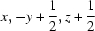; (iii) 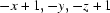.

**Fig. 1.**
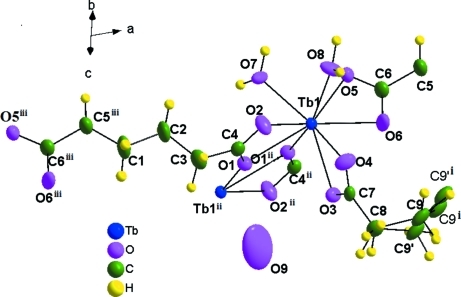
A view of part of the title structure. Ellipsoids are drwan at the 50% probability level. Symmetry code: (i) 2 - x, -y, 2 - z (ii) 1 - x, -y, 1 - z; (iii) -1 + x, y, z.]

**Fig. 2.**
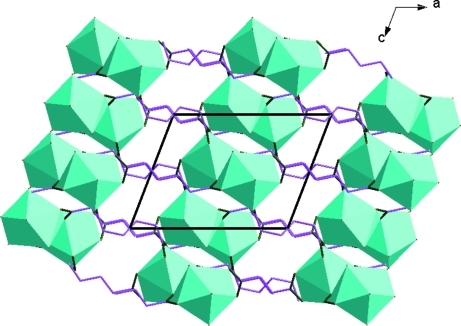
A view of the unit cell along the b-aixs of the title compound. showing TbO9 polyhedra and the adipate ligands (represented by lines).

## supplementary crystallographic information

### Comment

In recent years, a great interest has been focused on the crystal engineering of novel coordination polymers, not only due to their intriguing topological structures but also potential application as functional materials in areas such as ion exchange, catalysis, optics, gas separation/storage and sensing (Moulton & Zaworotko, 2001; Wood & Thompson, 2007). The *RE*-adipate (*RE* = rare earth metal) system has been examined extensively owing to the rich structural diversity of this family of materials. A great many of compounds have been reported which exhibit structure types ranging from 1-D chain to 2-D layer and 3-D framework topologies (Dimos *et al.*, 2002; Duan *et al.*, 2004; Kim *et al.*, 2004; Kiritsis *et al.*, 1998). Arguably much of this diversity is related to the flexibility of the aliphatic dicarboxylic backbone. In this paper, we report the hydrothermal synthesis and single-crystal X-ray diffraction analysis of a novel Tb-adipate compound, which is isotypic with La(III) (Kim *et al.*, 2004) and Dy(III) (Lill *et al.*, 2005) analogous complexes.

The crystal structure of the title complex consists of nine oxygen atoms coordinated to Tb(III) (Fig. 1) of which seven oxygen atoms are from four adipate ligands and two from two independent coordinated water molecules. Two symmetric TbO_9_ polyhedra share their edges to form a Tb_2_O_16_ dimeric unit about an inversion centet. These dimers are further linked through adipate anions to form a two-dimensional layer perpendicular to (010) (Fig. 2).

C9-atom of the adipate ligand was disordered over two sites with site occupancy factors 0.622 (9) and 0.378 (9). The structure also contains a disordered molecule of water of hydration lying close to a special position with partial occupancy.

### Experimental

Colorless prismatic single crystals of the title complex were obtained using hydrothermal methods in a sealed 20 ml Teflon-lined Parr bomb. TbCl_3_^.^ 6H_2_O (0.2 g), adipic acid (0.1 g) and H_2_O (10 ml) were placed in the bomb and sealed. The bomb was then heated under autogenous pressure for 7 d at 433 K and finally cooled to room temperature. Upon opening the bomb, a few single crystals was obtained for X-ray single-crystal diffraction analysis.

### Refinement

The H-atoms bonded to C-atoms were placed in calculated positions using a riding model, with C—H = 0.93–0.97 Å and *U*_iso_ = 1.2*U*_eq_. The H-atom of water molecules were located from the difference maps and fixed at those locations with *U*_iso_ = 1.5*U*_eq_(O).

### Crystal data

**Table:**

[Tb_2_(C_6_H_8_O_4_)_3_(H_2_O)_4_]·0.25H_2_O	*F*(000) = 801
*M**_r_* = 826.78	*D*_x_ = 2.036 Mg m^−^^3^
Monoclinic, *P*2_1_/*c*	Mo *K*α radiation, λ = 0.71073 Å
Hall symbol: -P 2ybc	Cell parameters from 5053 reflections
*a* = 11.603 (6) Å	θ = 2.4–28.4°
*b* = 13.886 (7) Å	µ = 5.27 mm^−^^1^
*c* = 8.969 (4) Å	*T* = 298 K
β = 111.017 (7)°	Prism, colourless
*V* = 1348.9 (11) Å^3^	0.25 × 0.05 × 0.05 mm
*Z* = 2	

### Data collection

**Table:**

Bruker APEXII CCD diffractometer	2335 independent reflections
Radiation source: fine-focus sealed tube	2008 reflections with *I* > 2σ(*I*)
graphite	*R*_int_ = 0.037
ω scans	θ_max_ = 25.0°, θ_min_ = 1.9°
Absorption correction: multi-scan (*SADABS*; Sheldrick, 1996)	*h* = −13→13
*T*_min_ = 0.352, *T*_max_ = 0.779	*k* = −16→16
7908 measured reflections	*l* = −10→10

### Refinement

**Table:**

Refinement on *F*^2^	Primary atom site location: structure-invariant direct methods
Least-squares matrix: full	Secondary atom site location: difference Fourier map
*R*[*F*^2^ > 2σ(*F*^2^)] = 0.025	Hydrogen site location: inferred from neighbouring sites
*wR*(*F*^2^) = 0.066	H-atom parameters constrained
*S* = 1.06	*w* = 1/[σ^2^(*F*_o_^2^) + (0.0379*P*)^2^] where *P* = (*F*_o_^2^ + 2*F*_c_^2^)/3
2335 reflections	(Δ/σ)_max_ = 0.001
176 parameters	Δρ_max_ = 0.89 e Å^−^^3^
6 restraints	Δρ_min_ = −1.84 e Å^−^^3^

### Special details

**Table:**

Geometry. All e.s.d.'s (except the e.s.d. in the dihedral angle between two l.s. planes) are estimated using the full covariance matrix. The cell e.s.d.'s are taken into account individually in the estimation of e.s.d.'s in distances, angles and torsion angles; correlations between e.s.d.'s in cell parameters are only used when they are defined by crystal symmetry. An approximate (isotropic) treatment of cell e.s.d.'s is used for estimating e.s.d.'s involving l.s. planes.
Refinement. Refinement of *F*^2^ against ALL reflections. The weighted *R*-factor *wR* and goodness of fit *S* are based on *F*^2^, conventional *R*-factors *R* are based on *F*, with *F* set to zero for negative *F*^2^. The threshold expression of *F*^2^ > σ(*F*^2^) is used only for calculating *R*-factors(gt) *etc*. and is not relevant to the choice of reflections for refinement. *R*-factors based on *F*^2^ are statistically about twice as large as those based on *F*, and *R*- factors based on ALL data will be even larger.

### Fractional atomic coordinates and isotropic or equivalent isotropic displacement parameters (Å^2^)

**Table:**

	*x*	*y*	*z*	*U*_iso_*/*U*_eq_	Occ. (<1)
Tb1	0.629324 (17)	0.108556 (13)	0.55754 (2)	0.01822 (10)	
O1	0.4353 (3)	0.0374 (2)	0.5851 (3)	0.0239 (7)	
O2	0.4828 (3)	0.1849 (2)	0.6661 (4)	0.0350 (8)	
O3	0.7081 (3)	−0.0211 (2)	0.7500 (4)	0.0320 (8)	
O4	0.7368 (4)	0.1232 (2)	0.8498 (4)	0.0352 (8)	
O5	0.7300 (3)	0.1371 (2)	0.3584 (4)	0.0277 (7)	
O6	0.8450 (3)	0.0963 (3)	0.5998 (5)	0.0453 (10)	
O7	0.4652 (3)	0.1591 (2)	0.3279 (4)	0.0354 (8)	
H1	0.4773	0.2153	0.2830	0.053*	
H2	0.4046	0.1137	0.2594	0.053*	
O8	0.6789 (3)	0.2728 (2)	0.5763 (4)	0.0375 (8)	
H3	0.6884	0.3209	0.5031	0.056*	
H4	0.7033	0.3077	0.6696	0.056*	
C1	0.0666 (4)	0.1439 (5)	0.5747 (7)	0.0464 (14)	
H1A	0.0643	0.0808	0.6199	0.056*	
H1B	0.0690	0.1913	0.6552	0.056*	
C2	0.1850 (4)	0.1522 (4)	0.5390 (7)	0.0398 (13)	
H2A	0.1790	0.1117	0.4484	0.048*	
H2B	0.1957	0.2183	0.5113	0.048*	
C3	0.2943 (5)	0.1221 (4)	0.6808 (7)	0.0349 (12)	
H3A	0.2774	0.0600	0.7181	0.042*	
H3B	0.3067	0.1683	0.7663	0.042*	
C4	0.4110 (4)	0.1151 (3)	0.6439 (6)	0.0242 (10)	
C5	0.9490 (4)	0.1579 (4)	0.4326 (6)	0.0351 (12)	
H5A	0.9417	0.2252	0.4013	0.042*	
H5B	0.9534	0.1203	0.3437	0.042*	
C6	0.8359 (5)	0.1287 (3)	0.4661 (6)	0.0286 (11)	
C7	0.7559 (4)	0.0338 (3)	0.8668 (5)	0.0242 (10)	
C8	0.8394 (4)	−0.0062 (4)	1.0230 (6)	0.0373 (12)	
H8A	0.8330	−0.0759	1.0201	0.045*	0.622 (9)
H8B	0.8127	0.0168	1.1074	0.045*	0.622 (9)
H8A'	0.8000	−0.0608	1.0522	0.045*	0.378 (9)
H8B'	0.8548	0.0424	1.1049	0.045*	0.378 (9)
C9	0.9742 (8)	0.0224 (7)	1.0609 (10)	0.0380 (18)	0.622 (9)
H9A	1.0234	0.0009	1.1677	0.046*	0.622 (9)
H9B	0.9804	0.0920	1.0582	0.046*	0.622 (9)
C9'	0.9608 (13)	−0.0416 (11)	1.0113 (17)	0.0380 (18)	0.378 (9)
H9'1	1.0072	−0.0765	1.1078	0.046*	0.378 (9)
H9'2	0.9429	−0.0857	0.9219	0.046*	0.378 (9)
H1O9	0.4920	0.0730	−0.0380	0.046*	0.125
H2O9	0.5400	0.0476	0.1203	0.046*	0.125
O9	0.508 (4)	0.024 (2)	0.026 (5)	0.063 (10)	0.125

### Atomic displacement parameters (Å^2^)

**Table:**

	*U*^11^	*U*^22^	*U*^33^	*U*^12^	*U*^13^	*U*^23^
Tb1	0.01601 (14)	0.02031 (14)	0.01799 (15)	−0.00133 (8)	0.00566 (10)	0.00041 (8)
O1	0.0267 (16)	0.0223 (15)	0.0254 (18)	0.0007 (14)	0.0126 (14)	−0.0019 (13)
O2	0.0334 (18)	0.0273 (17)	0.051 (2)	−0.0085 (15)	0.0226 (18)	−0.0127 (16)
O3	0.0277 (17)	0.0314 (17)	0.029 (2)	−0.0065 (15)	0.0006 (16)	0.0036 (15)
O4	0.049 (2)	0.0325 (18)	0.0229 (19)	0.0097 (17)	0.0118 (18)	−0.0019 (14)
O5	0.0216 (17)	0.0349 (16)	0.0253 (19)	−0.0041 (14)	0.0069 (15)	0.0031 (14)
O6	0.0211 (18)	0.078 (3)	0.040 (2)	0.0062 (17)	0.0150 (17)	0.0262 (19)
O7	0.0312 (18)	0.0276 (17)	0.035 (2)	−0.0052 (15)	−0.0035 (16)	0.0118 (15)
O8	0.063 (2)	0.0265 (16)	0.028 (2)	−0.0183 (17)	0.0217 (18)	−0.0066 (14)
C1	0.021 (3)	0.074 (4)	0.046 (4)	−0.005 (3)	0.015 (3)	−0.002 (3)
C2	0.020 (2)	0.054 (3)	0.051 (4)	−0.002 (2)	0.019 (3)	−0.001 (3)
C3	0.030 (3)	0.042 (3)	0.039 (3)	−0.003 (2)	0.020 (3)	−0.006 (2)
C4	0.023 (2)	0.031 (3)	0.018 (3)	0.003 (2)	0.005 (2)	0.0017 (18)
C5	0.023 (2)	0.048 (3)	0.036 (3)	−0.003 (2)	0.012 (2)	0.009 (2)
C6	0.025 (3)	0.035 (3)	0.029 (3)	0.001 (2)	0.014 (2)	0.000 (2)
C7	0.015 (2)	0.035 (3)	0.022 (3)	0.000 (2)	0.006 (2)	0.003 (2)
C8	0.034 (3)	0.050 (3)	0.027 (3)	0.003 (2)	0.009 (2)	0.010 (2)
C9	0.035 (4)	0.044 (5)	0.026 (5)	0.015 (4)	−0.001 (3)	0.005 (4)
C9'	0.035 (4)	0.044 (5)	0.026 (5)	0.015 (4)	−0.001 (3)	0.005 (4)
O9	0.062 (12)	0.065 (14)	0.057 (13)	−0.001 (10)	0.014 (9)	0.000 (9)

### Geometric parameters (Å, °)

**Table:**

Tb1—O8	2.344 (3)	C2—C3	1.498 (7)
Tb1—O7	2.355 (3)	C2—H2A	0.9700
Tb1—O1^i^	2.371 (3)	C2—H2B	0.9700
Tb1—O6	2.398 (4)	C3—C4	1.508 (7)
Tb1—O3	2.435 (3)	C3—H3A	0.9700
Tb1—O4	2.474 (4)	C3—H3B	0.9700
Tb1—O2	2.479 (3)	C5—C6	1.503 (6)
Tb1—O5	2.492 (3)	C5—C1^iii^	1.509 (7)
Tb1—O1	2.550 (3)	C5—H5A	0.9700
Tb1—C6	2.812 (5)	C5—H5B	0.9700
Tb1—C7	2.830 (4)	C7—C8	1.495 (6)
Tb1—C4	2.904 (5)	C8—C9'	1.530 (15)
O1—C4	1.275 (5)	C8—C9	1.530 (10)
O1—Tb1^i^	2.371 (3)	C8—H8A	0.9700
O2—C4	1.247 (5)	C8—H8B	0.9700
O3—C7	1.252 (5)	C8—H8A'	0.9684
O4—C7	1.261 (5)	C8—H8B'	0.9664
O5—C6	1.267 (6)	C9—C9^iv^	1.551 (17)
O6—C6	1.249 (6)	C9—H9A	0.9700
O7—H1	0.9127	C9—H9B	0.9700
O7—H2	0.9795	C9'—C9'^iv^	1.53 (3)
O8—H3	0.9699	C9'—H9'1	0.9700
O8—H4	0.9193	C9'—H9'2	0.9700
C1—C5^ii^	1.509 (7)	O9—O9^v^	0.80 (6)
C1—C2	1.523 (7)	O9—H1O9	0.8634
C1—H1A	0.9700	O9—H2O9	0.8563
C1—H1B	0.9700		
			
O8—Tb1—O7	82.75 (12)	H3—O8—H4	100.4
O8—Tb1—O1^i^	153.26 (10)	C5^ii^—C1—C2	115.0 (5)
O7—Tb1—O1^i^	77.45 (11)	C5^ii^—C1—H1A	108.5
O8—Tb1—O6	80.95 (13)	C2—C1—H1A	108.5
O7—Tb1—O6	129.04 (12)	C5^ii^—C1—H1B	108.5
O1^i^—Tb1—O6	97.73 (12)	C2—C1—H1B	108.5
O8—Tb1—O3	130.46 (11)	H1A—C1—H1B	107.5
O7—Tb1—O3	145.14 (10)	C3—C2—C1	110.7 (5)
O1^i^—Tb1—O3	73.49 (11)	C3—C2—H2A	109.5
O6—Tb1—O3	74.36 (11)	C1—C2—H2A	109.5
O8—Tb1—O4	80.00 (11)	C3—C2—H2B	109.5
O7—Tb1—O4	147.57 (12)	C1—C2—H2B	109.5
O1^i^—Tb1—O4	125.71 (10)	H2A—C2—H2B	108.1
O6—Tb1—O4	74.80 (13)	C2—C3—C4	112.7 (4)
O3—Tb1—O4	52.50 (11)	C2—C3—H3A	109.1
O8—Tb1—O2	74.98 (11)	C4—C3—H3A	109.1
O7—Tb1—O2	76.28 (12)	C2—C3—H3B	109.1
O1^i^—Tb1—O2	116.67 (10)	C4—C3—H3B	109.1
O6—Tb1—O2	142.34 (13)	H3A—C3—H3B	107.8
O3—Tb1—O2	100.00 (12)	O2—C4—O1	119.3 (4)
O4—Tb1—O2	72.83 (12)	O2—C4—C3	121.1 (4)
O8—Tb1—O5	74.37 (10)	O1—C4—C3	119.6 (4)
O7—Tb1—O5	76.46 (11)	O2—C4—Tb1	58.0 (2)
O1^i^—Tb1—O5	83.58 (10)	O1—C4—Tb1	61.3 (2)
O6—Tb1—O5	52.68 (11)	C3—C4—Tb1	176.8 (3)
O3—Tb1—O5	118.21 (11)	C6—C5—C1^iii^	112.7 (4)
O4—Tb1—O5	123.96 (12)	C6—C5—H5A	109.1
O2—Tb1—O5	141.00 (11)	C1^iii^—C5—H5A	109.1
O8—Tb1—O1	124.91 (11)	C6—C5—H5B	109.1
O7—Tb1—O1	74.63 (11)	C1^iii^—C5—H5B	109.1
O1^i^—Tb1—O1	66.54 (11)	H5A—C5—H5B	107.8
O6—Tb1—O1	149.81 (10)	O6—C6—O5	119.3 (4)
O3—Tb1—O1	76.40 (10)	O6—C6—C5	120.7 (5)
O4—Tb1—O1	93.22 (11)	O5—C6—C5	120.0 (4)
O2—Tb1—O1	51.26 (10)	O6—C6—Tb1	58.1 (2)
O5—Tb1—O1	142.02 (10)	O5—C6—Tb1	62.4 (2)
O8—Tb1—C6	73.22 (13)	C5—C6—Tb1	169.0 (4)
O7—Tb1—C6	102.82 (13)	O3—C7—O4	119.5 (4)
O1^i^—Tb1—C6	93.75 (12)	O3—C7—C8	120.1 (4)
O6—Tb1—C6	26.24 (13)	O4—C7—C8	120.4 (4)
O3—Tb1—C6	97.93 (13)	O3—C7—Tb1	59.0 (2)
O4—Tb1—C6	98.19 (14)	O4—C7—Tb1	60.9 (2)
O2—Tb1—C6	148.01 (12)	C8—C7—Tb1	171.4 (3)
O5—Tb1—C6	26.77 (12)	C7—C8—C9'	111.0 (6)
O1—Tb1—C6	160.27 (12)	C7—C8—C9	112.3 (5)
O8—Tb1—C7	105.01 (12)	C9'—C8—C9	37.4 (6)
O7—Tb1—C7	159.89 (12)	C7—C8—H8A	109.1
O1^i^—Tb1—C7	99.65 (12)	C9'—C8—H8A	75.1
O6—Tb1—C7	70.97 (12)	C9—C8—H8A	109.1
O3—Tb1—C7	26.17 (11)	C7—C8—H8B	109.1
O4—Tb1—C7	26.44 (11)	C9'—C8—H8B	136.0
O2—Tb1—C7	87.70 (12)	C9—C8—H8B	109.1
O5—Tb1—C7	123.31 (11)	H8A—C8—H8B	107.9
O1—Tb1—C7	85.91 (11)	C7—C8—H8A'	109.5
C6—Tb1—C7	97.21 (13)	C9'—C8—H8A'	107.6
O8—Tb1—C4	99.53 (12)	C9—C8—H8A'	133.4
O7—Tb1—C4	73.43 (13)	H8A—C8—H8A'	35.6
O1^i^—Tb1—C4	91.91 (11)	H8B—C8—H8A'	74.5
O6—Tb1—C4	157.00 (13)	C7—C8—H8B'	109.5
O3—Tb1—C4	88.60 (12)	C9'—C8—H8B'	110.6
O4—Tb1—C4	82.60 (13)	C9—C8—H8B'	75.7
O2—Tb1—C4	25.24 (10)	H8A—C8—H8B'	135.1
O5—Tb1—C4	149.81 (12)	H8B—C8—H8B'	36.8
O1—Tb1—C4	26.02 (10)	H8A'—C8—H8B'	108.5
C6—Tb1—C4	172.36 (13)	C8—C9—C9^iv^	111.2 (9)
C7—Tb1—C4	86.88 (13)	C8—C9—H9A	109.4
C4—O1—Tb1^i^	150.7 (3)	C9^iv^—C9—H9A	109.4
C4—O1—Tb1	92.7 (3)	C8—C9—H9B	109.4
Tb1^i^—O1—Tb1	113.46 (11)	C9^iv^—C9—H9B	109.4
C4—O2—Tb1	96.8 (3)	H9A—C9—H9B	108.0
C7—O3—Tb1	94.8 (3)	C9'^iv^—C9'—C8	112.0 (14)
C7—O4—Tb1	92.7 (3)	C9'^iv^—C9'—H9'1	109.2
C6—O5—Tb1	90.8 (3)	C8—C9'—H9'1	109.2
C6—O6—Tb1	95.7 (3)	C9'^iv^—C9'—H9'2	109.2
Tb1—O7—H1	115.9	C8—C9'—H9'2	109.2
Tb1—O7—H2	122.0	H9'1—C9'—H9'2	107.9
H1—O7—H2	117.6	O9^v^—O9—H1O9	108.8
Tb1—O8—H3	134.9	O9^v^—O9—H2O9	145.0
Tb1—O8—H4	124.4	H1O9—O9—H2O9	105.9
			
O8—Tb1—O1—C4	−14.2 (3)	O1^i^—Tb1—C4—O2	−169.2 (3)
O7—Tb1—O1—C4	−83.6 (3)	O6—Tb1—C4—O2	75.8 (4)
O1^i^—Tb1—O1—C4	−166.4 (3)	O3—Tb1—C4—O2	117.4 (3)
O6—Tb1—O1—C4	130.7 (3)	O4—Tb1—C4—O2	65.0 (3)
O3—Tb1—O1—C4	116.0 (3)	O5—Tb1—C4—O2	−88.7 (4)
O4—Tb1—O1—C4	65.8 (3)	O1—Tb1—C4—O2	178.3 (5)
O2—Tb1—O1—C4	0.9 (2)	C7—Tb1—C4—O2	91.2 (3)
O5—Tb1—O1—C4	−125.3 (3)	O8—Tb1—C4—O1	168.2 (2)
C6—Tb1—O1—C4	−168.7 (3)	O7—Tb1—C4—O1	88.8 (3)
C7—Tb1—O1—C4	91.3 (3)	O1^i^—Tb1—C4—O1	12.5 (3)
O8—Tb1—O1—Tb1^i^	152.16 (12)	O6—Tb1—C4—O1	−102.5 (4)
O7—Tb1—O1—Tb1^i^	82.74 (14)	O3—Tb1—C4—O1	−60.9 (2)
O1^i^—Tb1—O1—Tb1^i^	0.0	O4—Tb1—C4—O1	−113.3 (3)
O6—Tb1—O1—Tb1^i^	−63.0 (3)	O2—Tb1—C4—O1	−178.3 (5)
O3—Tb1—O1—Tb1^i^	−77.66 (14)	O5—Tb1—C4—O1	93.0 (3)
O4—Tb1—O1—Tb1^i^	−127.80 (12)	C7—Tb1—C4—O1	−87.1 (3)
O2—Tb1—O1—Tb1^i^	167.3 (2)	Tb1—O6—C6—O5	−12.6 (5)
O5—Tb1—O1—Tb1^i^	41.1 (2)	Tb1—O6—C6—C5	167.2 (4)
C6—Tb1—O1—Tb1^i^	−2.4 (4)	Tb1—O5—C6—O6	12.0 (5)
C7—Tb1—O1—Tb1^i^	−102.37 (14)	Tb1—O5—C6—C5	−167.8 (4)
C4—Tb1—O1—Tb1^i^	166.4 (3)	C1^iii^—C5—C6—O6	−0.4 (7)
O8—Tb1—O2—C4	166.2 (3)	C1^iii^—C5—C6—O5	179.4 (5)
O7—Tb1—O2—C4	80.2 (3)	C1^iii^—C5—C6—Tb1	79.9 (19)
O1^i^—Tb1—O2—C4	12.1 (3)	O8—Tb1—C6—O6	103.9 (3)
O6—Tb1—O2—C4	−141.7 (3)	O7—Tb1—C6—O6	−177.9 (3)
O3—Tb1—O2—C4	−64.4 (3)	O1^i^—Tb1—C6—O6	−99.9 (3)
O4—Tb1—O2—C4	−109.8 (3)	O3—Tb1—C6—O6	−26.1 (3)
O5—Tb1—O2—C4	127.0 (3)	O4—Tb1—C6—O6	27.0 (3)
O1—Tb1—O2—C4	−1.0 (3)	O2—Tb1—C6—O6	97.6 (4)
C6—Tb1—O2—C4	172.5 (3)	O5—Tb1—C6—O6	−167.6 (5)
C7—Tb1—O2—C4	−87.6 (3)	O1—Tb1—C6—O6	−97.7 (4)
O8—Tb1—O3—C7	15.6 (3)	C7—Tb1—C6—O6	0.3 (3)
O7—Tb1—O3—C7	−143.5 (3)	O8—Tb1—C6—O5	−88.5 (3)
O1^i^—Tb1—O3—C7	−178.2 (3)	O7—Tb1—C6—O5	−10.2 (3)
O6—Tb1—O3—C7	78.6 (3)	O1^i^—Tb1—C6—O5	67.7 (3)
O4—Tb1—O3—C7	−4.0 (2)	O6—Tb1—C6—O5	167.6 (5)
O2—Tb1—O3—C7	−63.2 (3)	O3—Tb1—C6—O5	141.6 (3)
O5—Tb1—O3—C7	108.7 (3)	O4—Tb1—C6—O5	−165.4 (3)
O1—Tb1—O3—C7	−109.0 (3)	O2—Tb1—C6—O5	−94.8 (3)
C6—Tb1—O3—C7	90.2 (3)	O1—Tb1—C6—O5	69.9 (5)
C4—Tb1—O3—C7	−85.8 (3)	C7—Tb1—C6—O5	168.0 (3)
O8—Tb1—O4—C7	−161.0 (3)	O8—Tb1—C6—C5	17.0 (18)
O7—Tb1—O4—C7	140.1 (3)	O7—Tb1—C6—C5	95.2 (19)
O1^i^—Tb1—O4—C7	10.9 (3)	O1^i^—Tb1—C6—C5	173.2 (19)
O6—Tb1—O4—C7	−77.7 (3)	O6—Tb1—C6—C5	−86.9 (19)
O3—Tb1—O4—C7	4.0 (2)	O3—Tb1—C6—C5	−113.0 (19)
O2—Tb1—O4—C7	121.8 (3)	O4—Tb1—C6—C5	−59.9 (19)
O5—Tb1—O4—C7	−97.6 (3)	O2—Tb1—C6—C5	11 (2)
O1—Tb1—O4—C7	74.1 (3)	O5—Tb1—C6—C5	105.5 (19)
C6—Tb1—O4—C7	−89.7 (3)	O1—Tb1—C6—C5	175.4 (17)
C4—Tb1—O4—C7	97.9 (3)	C7—Tb1—C6—C5	−86.6 (19)
O8—Tb1—O5—C6	83.6 (3)	Tb1—O3—C7—O4	7.2 (4)
O7—Tb1—O5—C6	169.7 (3)	Tb1—O3—C7—C8	−170.0 (3)
O1^i^—Tb1—O5—C6	−111.7 (3)	Tb1—O4—C7—O3	−7.1 (4)
O6—Tb1—O5—C6	−6.8 (3)	Tb1—O4—C7—C8	170.2 (4)
O3—Tb1—O5—C6	−44.3 (3)	O8—Tb1—C7—O3	−167.8 (2)
O4—Tb1—O5—C6	17.6 (3)	O7—Tb1—C7—O3	81.6 (4)
O2—Tb1—O5—C6	123.0 (3)	O1^i^—Tb1—C7—O3	1.8 (3)
O1—Tb1—O5—C6	−149.0 (3)	O6—Tb1—C7—O3	−93.2 (3)
C7—Tb1—O5—C6	−14.3 (3)	O4—Tb1—C7—O3	172.8 (4)
C4—Tb1—O5—C6	165.6 (3)	O2—Tb1—C7—O3	118.4 (3)
O8—Tb1—O6—C6	−70.2 (3)	O5—Tb1—C7—O3	−86.9 (3)
O7—Tb1—O6—C6	2.7 (4)	O1—Tb1—C7—O3	67.1 (2)
O1^i^—Tb1—O6—C6	82.8 (3)	C6—Tb1—C7—O3	−93.3 (3)
O3—Tb1—O6—C6	153.1 (3)	C4—Tb1—C7—O3	93.2 (3)
O4—Tb1—O6—C6	−152.3 (3)	O8—Tb1—C7—O4	19.4 (3)
O2—Tb1—O6—C6	−120.7 (3)	O7—Tb1—C7—O4	−91.2 (4)
O5—Tb1—O6—C6	7.0 (3)	O1^i^—Tb1—C7—O4	−171.0 (3)
O1—Tb1—O6—C6	138.3 (3)	O6—Tb1—C7—O4	94.1 (3)
C7—Tb1—O6—C6	−179.7 (3)	O3—Tb1—C7—O4	−172.8 (4)
C4—Tb1—O6—C6	−163.3 (3)	O2—Tb1—C7—O4	−54.4 (3)
C5^ii^—C1—C2—C3	−171.1 (5)	O5—Tb1—C7—O4	100.3 (3)
C1—C2—C3—C4	171.6 (4)	O1—Tb1—C7—O4	−105.7 (3)
Tb1—O2—C4—O1	1.7 (5)	C6—Tb1—C7—O4	93.9 (3)
Tb1—O2—C4—C3	−176.4 (4)	C4—Tb1—C7—O4	−79.6 (3)
Tb1^i^—O1—C4—O2	−155.4 (4)	O3—C7—C8—C9'	69.0 (8)
Tb1—O1—C4—O2	−1.7 (4)	O4—C7—C8—C9'	−108.2 (8)
Tb1^i^—O1—C4—C3	22.7 (8)	O3—C7—C8—C9	109.3 (6)
Tb1—O1—C4—C3	176.5 (4)	O4—C7—C8—C9	−67.9 (6)
Tb1^i^—O1—C4—Tb1	−153.8 (6)	C7—C8—C9—C9^iv^	−65.1 (10)
C2—C3—C4—O2	92.4 (6)	C9'—C8—C9—C9^iv^	30.7 (10)
C2—C3—C4—O1	−85.7 (5)	C7—C8—C9'—C9'^iv^	68.2 (15)
O8—Tb1—C4—O2	−13.5 (3)	C9—C8—C9'—C9'^iv^	−31.4 (10)
O7—Tb1—C4—O2	−92.9 (3)		

Symmetry codes: (i) −*x*+1, −*y*, −*z*+1; (ii) *x*−1, *y*, *z*; (iii) *x*+1, *y*, *z*; (iv) −*x*+2, −*y*, −*z*+2; (v) −*x*+1, −*y*, −*z*.

### Hydrogen-bond geometry (Å, °)

**Table:**

*D*—H···*A*	*D*—H	H···*A*	*D*···*A*	*D*—H···*A*
O8—H3···O4^vi^	0.97	1.83	2.764 (4)	160
O8—H4···O5^vii^	0.92	1.78	2.691 (4)	170
O7—H1···O2^vi^	0.91	1.75	2.657 (4)	170
O7—H2···O3^i^	0.98	1.81	2.682 (4)	146

Symmetry codes: (vi) *x*, −*y*+1/2, *z*−1/2; (vii) *x*, −*y*+1/2, *z*+1/2; (i) −*x*+1, −*y*, −*z*+1.
